# Sequential oxidative transformation of the chalcopyrite (001)-S surface by H$_{2}$O and O$_{2}$: electronic structure insights from DFT calculations

**DOI:** 10.1007/s00894-026-06972-5

**Published:** 2026-10-13

**Authors:** Guilherme Randow Nascimento, Selma Fabiana Bazan, Heitor Avelino de Abreu, Guilherme Ferreira de Lima

**Affiliations:** Department of Chemistry, Universidade Federal de Minas Gerais, Belo Horizonte, MG Brazil

**Keywords:** Chalcopyrite, DFT, Oxidation, Leaching

## Abstract

**Context:**

Chalcopyrite (CuFeS$_{2}$) is the primary copper mineral worldwide, yet its hydrometallurgical extraction remains severely hindered by slow leaching kinetics, commonly attributed to surface passivation. Understanding the atomistic events governing the early stages of chalcopyrite surface oxidation is essential for developing more efficient and sustainable copper extraction technologies. In this work, a four-step oxidative mechanism on the CuFeS$_{2}$ ${\textbf {(001)}}$-S surface was investigated and characterized electronically. O$_{2}$ co-adsorption with H$_{2}$O on Fe sites triggers charge transfer into antibonding $\boldsymbol{\uppi }^{\boldsymbol{*}}$ orbitals, activating O$_{2}$ into a superoxide-like intermediate. Subsequent proton transfer yields Fe-OOH and Fe-OH species, followed by the dissociative adsorption of a second H$_{2}$O molecule onto the S-S dimer, forming S$_{2}$OH intermediates accompanied by S-Cu bond elongation from ${\textbf {2.35}}$ to ${\textbf {3.62}}$ Å. In the final step, H$_{2}$O adsorption on copper drives significant interfacial charge redistribution, with electronic states near the Fermi level progressively localized on oxygenated intermediates. Overall, iron, copper, and sulfur play distinct but electronically coupled roles in the progressive oxidative transformation of the chalcopyrite surface, providing atomistic insight into the early stages of passivation under hydrometallurgical conditions.

**Methods:**

All calculations were performed within the DFT+U framework using the PBE functional and a Hubbard correction (${\textbf {U = 2}}$ eV for Fe$^{3+}$), as implemented in Quantum ESPRESSO (v7.3). The CuFeS$_{2}$ (001)-S surface was modeled as a periodic slab with a ${\textbf {15}}$ Å vacuum layer, employing ultrasoft pseudopotentials and a plane-wave kinetic energy cutoff of ${\textbf {60}}$ Ry. Brillouin zone sampling used ${\textbf {1}}\boldsymbol{\times }  {\textbf {1}}\boldsymbol{\times }  {\textbf {1}}$ and ${\textbf {4}}\boldsymbol{\times }  {\textbf {4}}\boldsymbol{\times }  {\textbf {1}}$ ${\boldsymbol{k}}$-point grids for geometry optimizations and electronic property calculations, respectively. Antiferromagnetic ordering along the crystallographic *c*-axis was adopted throughout. Electronic structure characterization was performed through Bader charge analysis, projected density of states (PDOS), charge density difference maps, and integrated local density of states (ILDOS).

**Graphical abstract:**

**Figure:**
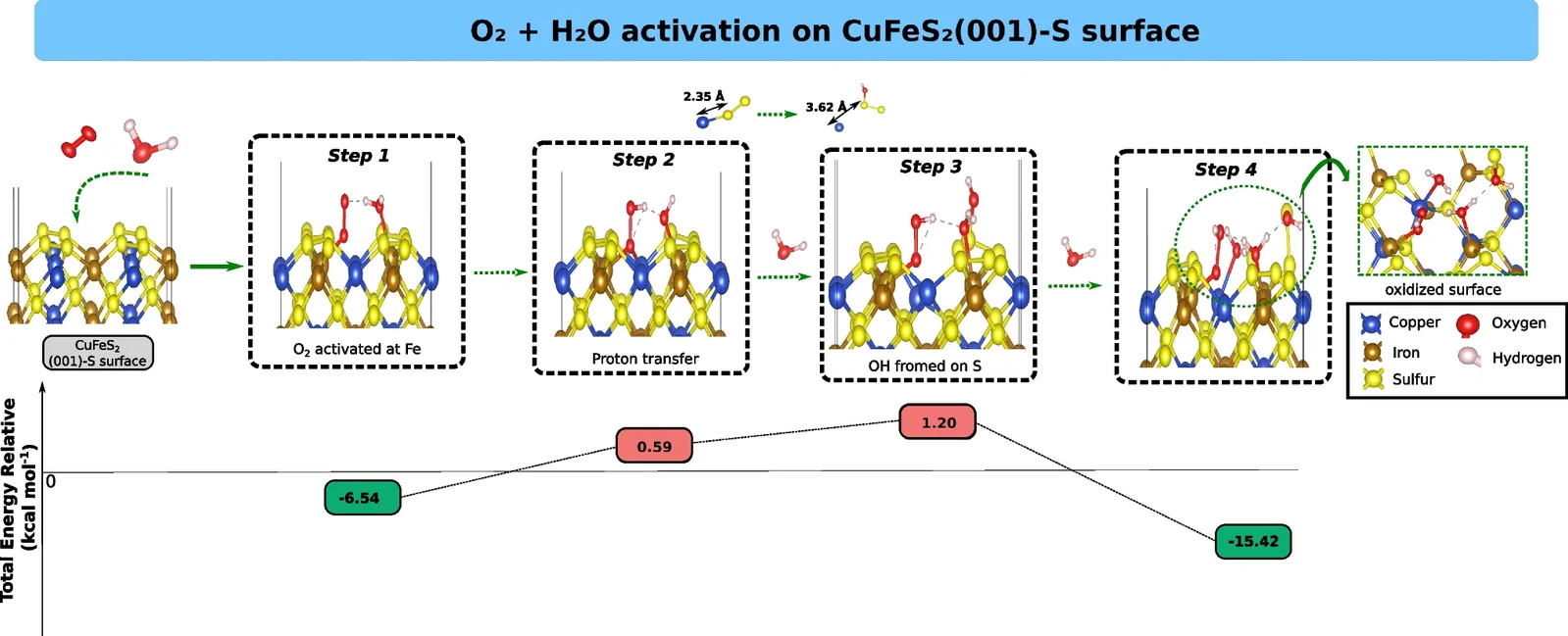

## Introduction

The escalation of environmental issues, such as the greenhouse effect and global warming, has significantly impacted production methods and livelihoods [1]. Consequently, global economies are seeking alternatives to mitigate these environmental impacts, primarily through decarbonization and the transformation of the energy matrix. In this context, mineral resources are essential for the energy transition, as they are crucial for producing energy from renewable sources, developing infrastructure, and transitioning from fossil-fuel-based vehicles to electric or renewable energy systems [2–4]. The demand for mineral resources is projected to grow steadily over the coming years [5–7]. Among these critical minerals, copper stands out due to its versatility and its central role in virtually every technology underpinning the energy transition. It is essential for numerous applications, such as the construction of wind power plants and electrical transmission wiring [8, 9]. In civil construction and building infrastructure, copper is indispensable for electrical transmission wiring [8, 9], plumbing systems, roofing, and HVAC (Heating, Ventilation, and Air Conditioning) installations [10, 11]. Indeed, building construction represents the single largest end-use of copper globally, accounting for approximately 42% of all copper consumed in the United States in 2024 alone [12]. Beyond construction, copper is a critical material for electric vehicles (EVs); while a conventional car contains an average of 23 kg of Cu, hybrid, plug-in, and battery electric vehicles require 40 kg, 60 kg, and 83 kg of Cu per vehicle, respectively [13]. Beyond its direct usage, copper mining dynamics are also crucial for battery supply chains, as the availability of essential elements like cobalt is highly dependent on regional and global copper extraction [13]. With the ongoing shift in the energy matrix, a significant surge in copper demand is anticipated.

Recent studies indicate that the demand for mined copper is projected to reach 37.1 Mtpy (million tons per year) by 2050. To meet this demand, an estimated 1,104 Mt of copper will need to be extracted between 2018 and 2050, representing a 115% increase compared to the period from 1900 to 2018. For comparison, global copper production increased from 0.5 Mt in 1900 to 20.4 Mt in 2018 [14]. These projections underscore the urgent need to develop more efficient and sustainable extraction technologies capable of meeting future demand.

Satisfying this demand solely through primary mining remains a significant challenge, as copper is typically extracted from chalcopyrite via two main routes: pyrometallurgical and hydrometallurgical. The choice between these processes depends largely on the ore grade; while high-grade ores are traditionally treated through pyrometallurgy, low-grade ores are better suited for hydrometallurgical processing. As high-concentration deposits become increasingly scarce, optimizing processes capable of handling low-grade ores becomes essential to ensure future supply [15].

Chalcopyrite (CuFeS$_{2}$) is the most abundant copper mineral [16], crystallizing in the tetragonal system with the space group $I\bar{4}2d$. According to X-ray diffraction studies by Burdick and Ellis [17], its lattice parameters are *a*=5.289 Å and **c**=10.423 Å. In this structure, copper and iron atoms are tetrahedrally coordinated by four sulfur atoms, with formal oxidation states of Cu$^{+}$, Fe${^{3+}}$, and S${^{2-}}$, as corroborated by spectroscopic studies [18, 19], including X-ray photoelectron spectroscopy (XPS) and X-ray absorption spectroscopy (XAS), as well as by first-principles calculations [20]. Furthermore, chalcopyrite is characterized as an antiferromagnetic semiconductor, with iron ions exhibiting opposite spins along the crystallographic c-axis and a bandgap of approximately 0.5 eV [21, 22].

Despite its status as the most significant copper mineral, the extraction of chalcopyrite involves complex metallurgical challenges. Pyrometallurgy is currently the main extraction route, accounting for approximately 80% of global production. However, this process faces significant limitations, such as the generation of hazardous sulfur dioxide (SO$_{2}$) emissions, the requirement of high operating temperatures, and the dependence on high-grade ores to remain economically viable. A potential alternative is the hydrometallurgical route, which primarily involves acid leaching after a concentration step. Although hydrometallurgy is generally considered more cost-effective and environmentally favorable for low-grade ores, its application to chalcopyrite is hindered by extremely slow leaching kinetics [16, 23].

The hydrometallurgical route is fundamentally based on leaching and typically comprises three main stages: leaching, solvent extraction, and electrowinning [24]. The leaching step generally occurs in acidic solutions. However, due to the refractory nature of chalcopyrite, elevated temperatures are often required to overcome the kinetic barriers imposed by its refractory nature and allow copper to be solubilized. This refractoriness arises from two intrinsic properties of the mineral: its exceptionally high lattice energy (17,500 kJ mol$^{-1}$ ), which is four to six times greater than that of other common sulfide minerals, and its semiconducting character, with a band gap of approximately 0.5–0.6 eV that restricts electron transfer at the mineral–solution interface, thereby limiting the oxidation kinetics [23, 25]. Under oxidative conditions, the dissolution of chalcopyrite can be described by Eqs. 1 through 4 [16].

1 $$\begin{aligned} \text {2}\,\text {CuFeS}_{2}\, \text {(s)}+ &   \text {4}\,\text {Fe}_{2}\text {(SO}_{4}\text {)}_{3} \text {(aq)} \longrightarrow \text {2}\,\text {Cu}{^{2+}} \text {(aq) + 2}\,\text {SO}_{4}{}^{2-} \text {(aq)} \nonumber \\+ &   \text {10}\,\text {FeSO}_{4} \text {(aq) + 4}\,\text {S} \,\text {(s)} \end{aligned}$$

2 $$\begin{aligned} {\text {4}\,\text {FeSO}_{4}\, \text {(aq)} + \text {O}_{2}\, \text {(g)} + \text {2}\,\text {H}_{2}\text {SO}_{4}\, \text {(aq)} \longrightarrow \text {2}\,\text {Fe}_{2}\text {(SO}_{4}\text {)}_{3}\, \text {(aq)} + \text {2}\,\text {H}_{2}\text {O}\, \text {(l)}} \end{aligned}$$

3 $$\begin{aligned} {\text {2}\,\text {S} \,\text {(s)} + \text {3}\,\text {O}_{2}\, \text {(g)} + \text {2}\,\text {H}_{2}\text {O} \,\text {(l)} \longrightarrow \text {2}\,\text {H}_{2}\text {SO}_{4}\, \text {(aq)}} \end{aligned}$$

4 $$\begin{aligned} \text {4}\,\text {CuFeS}_{2}\, \text {(s)}+ &   \text {17}\,\text {O}_{2}\, \text {(g)} + \text {4}\,\text {H}_{2}\text {O}\, \text {(l)} \longrightarrow \text {4}\,\text {Cu}{^{2+}}\, \text {(aq)} + \text {4}\,\text {SO}_{4}{}^{{2-}}\, \text {(aq)} \nonumber \\+ &   \text {2}\,\text {Fe}_{2}\text {O}_{3}\, \text {(s)} + \text {4}\,\text {H}_{2}\text {SO}_{4}\, \text {(aq)} \end{aligned}$$

Understanding the leaching mechanism is essential to developing more efficient and sustainable methods. In this context, oxidation plays a critical role, as the layers formed can inhibit subsequent leaching. Several studies using XPS, AES, and various spectroscopies [19, 26–30] reveal that oxidation behavior varies depending on the environment, such as air or aqueous media. Typically, species such as iron hydroxides and oxyhydroxides are initially formed, followed by sulfates and copper compounds, including FeOOH, Cu$_{2}$O, Cu$_{2}$S, and different sulfate species [23]. Recent studies using synchrotron scanning photoemission microscopy (SPEM) [31] indicate that, following air exposure, sulfide compounds still predominate on the surface, whereas polysulfides and disulfides are detected in smaller proportions [23].

Several lixiviants have been studied for chalcopyrite leaching, including Fe$^{3+}$, H$_{2}$O$_{2}$, dichromate, O$_{3}$, S$_{2}$O$_{8}{}^{2-}$, NO$_{3}{}^{-}$, and Ag$^{+}$ [32]. Among these, Fe$^{3+}$ is the most widely used oxidizing agent due to its ability to enhance leaching efficiency, its relatively simple implementation in acidic media, and low price. The reaction mechanism involves the oxidation of chalcopyrite by Fe$^{3+}$, resulting in the release of Cu$^{2+}$ into solution while Fe$^{3+}$ is reduced to Fe$^{2+}$.

Although relatively high copper recovery rates are achieved during the initial stages of ferric leaching at room temperature, the reaction rate decreases abruptly, a behavior commonly attributed to surface passivation. Nevertheless, the nature of this passive layer remains under debate, with elemental sulfur [29, 33, 34], polysulfides [35, 36], metal-deficient phases [37], and jarosite [38–40] being the most frequently cited species responsible for the kinetic inhibition.

Factors such as pH and exposure time during leaching significantly affect the composition of surface layers. Sulfur species, including S$^{0}$, S$_{2}{}^{2-}$, and S$_{n}{}^{2-}$, alongside iron hydroxides and oxyhydroxides, are the primary components detected [35]. These processes typically result in iron-deficient and copper-rich polysulfide layers. Although the presence of Fe$^{3+}$ promotes the formation of these species, it also triggers passivation, which hinders copper recovery. Furthermore, several theoretical and experimental models suggest that iron is leached preferentially before copper, a phenomenon that directly influences the reaction kinetics [23]. These findings highlight the complexity of the chalcopyrite–solution interface and the need for a detailed atomistic understanding of the surface processes governing passivation.

Due to the challenges in understanding passivation during leaching, theoretical calculations have been employed to comprehend the atomistic behavior of chalcopyrite surfaces. Density Functional Theory (DFT) has been widely used to investigate surface reconstruction [41] and the thermodynamic stability of various crystallographic planes [42]. Since the cleavage of the bulk crystal creates different surfaces, structural rearrangements typically occur to minimize surface energy. For instance, de Oliveira et al. [41] investigated the reconstruction of chalcopyrite surfaces, suggesting the formation of sulfur dimers through an oxidative process involving S$_{2}{}^{2-}$ groups following the cleavage of the sulfur-terminated (001) surfaces, called (001)-S. During this process, the electrons released by the formation of disulfide groups are transferred to iron atoms, promoting the reduction of Fe$^{3+}$ to Fe$^{2+}$. These results demonstrate that surface reconstruction significantly influences the electronic structure and, consequently, the reactivity of chalcopyrite surfaces.

During leaching, oxidation reactions on the surface transform its composition and impact copper extraction [23, 43–47]. Several steps involving the adsorption of water, acids, and oxygen onto the surface occur, leading to the formation of important sub-products such as FeO(OH) [43, 45]. XPS and DFT studies have shown that Fe is preferentially oxidized over Cu and S [45, 48–50]. The formation of FeO(OH) is considered a key step in passivation, a process that forms a protective layer, hindering further oxidation and resulting in low copper recovery rates [23, 47, 51].

Both computational and experimental studies have been developed to understand these processes and the interactions between water, oxygen, acids, and chalcopyrite [43, 46, 47, 52–54]. Since oxidation begins at the iron atoms, forming oxides and oxy-hydroxides, it is crucial to consider these aspects. Consequently, recent models have focused on: (i) oxygen adsorption (dissociative or non-dissociative), (ii) water adsorption (dissociation and speciation), and (iii) the co-adsorption of oxygen and water on the surface.

DFT studies have consistently demonstrated that O$_{2}$ adsorption on chalcopyrite surfaces occurs preferentially on Fe sites rather than Cu sites, both on the (001) and (112) surfaces, regardless of whether the termination is metal- or sulfur-based [43, 45, 55]. Among the possible adsorption configurations — vertical and parallel — the parallel arrangement is systematically more favorable [43, 45]. Regarding dissociation, two pathways have been identified: a one-step route, thermodynamically favored on Fe sites, and a two-step route, kinetically favored on Cu sites [43, 45]. This distinction implies that the nature of the oxidized surface species depends critically on whether the process is governed by thermodynamics or kinetics [51].

Water adsorption follows a similar site preference: isolated H$_{2}$O molecules adsorb preferentially on Fe atoms in a non-dissociative configuration compared to Cu and S sites — a behavior attributed to the relative weakness of the S–H bond in comparison to the O–H bond [43, 51, 55, 56]. However, water alone is insufficient to promote the oxidation of S atoms on a clean chalcopyrite surface [45]. Under oxidative conditions, after initial surface oxidation, water and oxygen co-adsorb and dissociate on Fe sites [45]. Specifically, H$_{2}$O can dissociate into H and OH species, forming Fe–O bonds on the (001)-M surface. On the (112)-M surface, dissociation requires overcoming a higher energy barrier, ultimately yielding FeOOH and FeOH species — key intermediates in the passivation process [43, 51].

Beyond water and oxygen, the interaction of leaching acids with the chalcopyrite surface is also relevant to the passivation process. DFT studies on the reconstructed sulfur-terminated (001) surface have shown that species such as Cl$^{-}$, SO$_{4}{}^{2-}$, and HSO$_{4}{}^{-}$ preferentially adsorb on Fe sites, while protons interact with surface disulfides, promoting S–S bond breaking, which helps explain the low leaching kinetics associated with polysulfide and jarosite passivation layers [54].

Previous theoretical research has explored the design of new molecules interacting with the surface, as well as their structural and electronic properties [57–60], in addition to adsorption and oxidation processes [43, 47]. However, this field still lacks in-depth studies due to the inherent complexity of interactions on the chalcopyrite surface. Understanding its structure, the surface formation process, and its reactivity is of paramount importance, as these insights can enable the development of more efficient copper extraction methods. Despite these advances, the interplay between water and oxygen on the sulfur-terminated (001)-S surface remains insufficiently understood, particularly regarding the sequence of adsorption events and the early stages of oxidized species formation. Therefore, this work aims to elucidate the surface phenomena occurring on chalcopyrite. Initially, the adsorption mechanisms of water and molecular oxygen on the chalcopyrite (001)-S surface will be investigated using Density Functional Theory (DFT) calculations. The study will determine the preferential adsorption sites, the associated adsorption energies, and the formation of oxidized surface species. Beyond energetics, a detailed electronic structure analysis, including projected density of states (PDOS), Bader charge analysis, charge density difference, and local density of states (ILDOS), will be performed to elucidate the nature of the chemical bonding and the charge transfer mechanisms underlying the surface reactivity.

## Methodology

The CuFeS$$_{2}$$ surface was constructed based on the pre-optimized structure reported by Bazan et al. [53]. All calculations were performed within the framework of Density Functional Theory (DFT) using the Quantum ESPRESSO package (v7.3).[61] Exchange–correlation effects were described using the Perdew–Burke–Ernzerhof (PBE) functional [62]. Ultrasoft pseudopotentials were employed, with the following valence configurations: Cu ($$4s{^{1.5}} 3{d^{9.5}}$$), Fe ($$3s^{2} 3p^{6} 4s^{2}$$$$3d^{6}$$), S ($$3s^{2} 3p^{4}$$), O ($$2s^{2} 2p^{4}$$), and H ($$1s^{1}$$). The Kohn-Sham wave functions were expanded in a plane-wave with a kinetic energy cutoff of 60 Ry. To accurately describe the strongly correlated 3*d* electrons of Fe$$^{3+}$$, a Hubbard correction of U=2 eV was applied, following the approach of Dudarev et al. [63]. This correction is particularly relevant for Fe$$^{3+}$$, whose open-shell 3d configuration is known to be poorly described by standard GGA functionals in sulfide minerals [64].

The value of *U* was determined by evaluating the calculated band gap as a function of this parameter. In the absence of the Hubbard correction (U=0 eV), the system exhibits a band gap of 0.5 eV, in agreement with the experimental value. The same band gap is obtained for U=2 eV. However, for U=4 eV, the band gap increases to 0.7 eV, deviating from the experimental value. At U=6 eV, the system exhibits metallic behavior. Therefore, U=2 eV was adopted, as it provides the best description of the electronic properties of the system in comparison with the experimental results.

Evaluation of the possible magnetic configurations of chalcopyrite showed that antiferromagnetic ordering along the *c*-axis corresponds to the most energetically stable configuration. The antiferromagnetic configurations along the *a*- and *b*-axes, as well as the partially compensated configuration, exhibit energies approximately 5 kcal mol$^{-1}$ higher than that of the most stable state. In turn, the ferromagnetic configuration is about 10 kcal mol$^{-1}$ higher in energy. Therefore, the system was described with antiferromagnetic ordering along the *c*-axis, as this configuration has the lowest total energy and is consistent with the experimentally observed magnetic structure of chalcopyrite [65].

The adsorption of H$_{2}$O and O$_{2}$ molecules was investigated on the (001) surface of chalcopyrite, which was modeled as a periodic slab containing 64 atoms, with a lateral lattice parameter *a* = *b* = 10.69 Å and a cell height of *c* = 25.63 Å along the direction perpendicular to the surface. A vacuum layer of 15 Å was added perpendicular to the surface to prevent spurious interactions between periodic images. Brillouin zone sampling was performed using a $1\times 1\times 1$ **k**-point grid for structural optimizations, while a denser $4\times 4\times 1$ grid was employed for electronic property calculations. An antiferromagnetic ordering along the c-axis was adopted, consistent with the experimentally observed magnetic structure of chalcopyrite [65].

In the optimized surface model, H$_{2}$O and O$_{2}$ molecules were adsorbed by systematically exploring multiple initial configurations, including top, bridge, and hollow sites, to identify the most stable adsorption geometries. The subsequent reaction steps were modeled through the sequential addition of water molecules, following the mechanistic path for the oxidative leaching of chalcopyrite. Geometry optimizations were carried out using the same computational protocol described above, with convergence thresholds of 1×10$^{-4}$ Ry for the total energy and $10^{-3}\,\mathrm {Ry\,Bohr^{-1}}$ for the atomic forces. To evaluate the binding energies, isolated molecules were calculated in a vacuum supercell with dimensions similar to those used in the slab model.

The binding energy ($\Delta E_{ads}$) was calculated according to Eq. 5, where $E_{surface+molecule}$ corresponds to the total energy of the chalcopyrite surface with the adsorbed molecules, and $E_{surface}$ and $E_{molecule}$ represent the total energy of the isolated surface and the isolated molecule, respectively. By convention, negative values of $\Delta E_{ads}$ indicate thermodynamically favorable adsorption.

5 $$\begin{aligned} \Delta E_{ads}=E_{surface+molecule} - (E_{surface} + E_{molecule}) \end{aligned}$$

For each adsorption stage, the most stable configuration was subjected to self-consistent field (SCF) calculations followed by comprehensive electronic structure analyzes, including projected density of states (PDOS), Bader charge analysis [66] and integrated local density of states (ILDOS). This approach enabled a detailed characterization of the electronic properties of the adsorption complexes, including the identification of charge transfer and electron redistribution processes at the chalcopyrite surface.

## Results

**Fig. 1:**
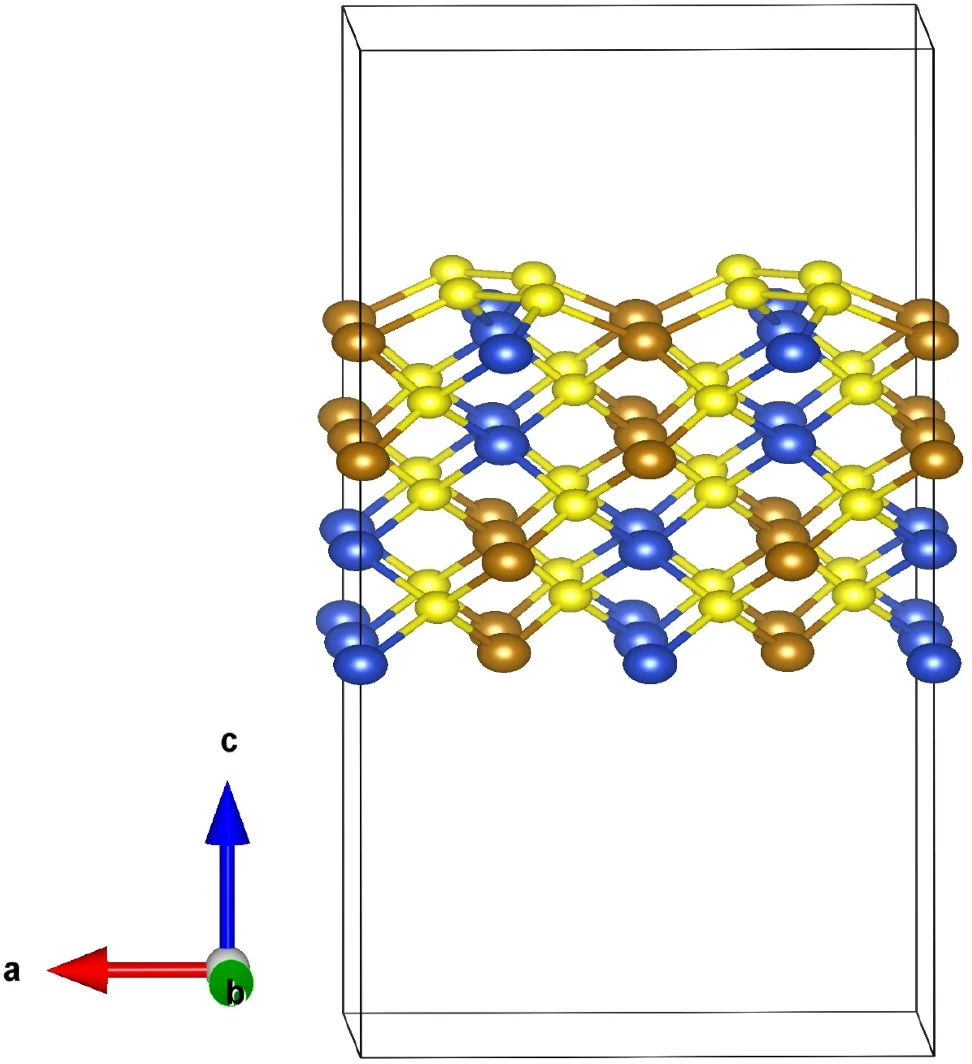
Optimized structure of the (001)–S chalcopyrite surface. Blue, brown, and yellow spheres represent copper, iron, and sulfur atoms, respectively

Among the crystallographic orientations of chalcopyrite, the (001) face is one of the most frequently exposed during comminution and exhibits high reactivity toward oxidation [42]. On this basis, the (001)-S termination was selected as the model surface. Figure 1 displays the surface after the optimization process, where sulfur dimers are visible, as previously reported by de Oliveira et al. [41]. The S–S, Cu–S, and Fe–S are 2.12, 2.35, and 2.37 Å, respectively, in good agreement with the values reported by de Oliveira et al. [41] (2.12, 2.30 and 2.30 Å). Small deviations observed for Cu–S, and Fe–S are attributed to differences in the computational protocol employed.

**Fig. 2:**
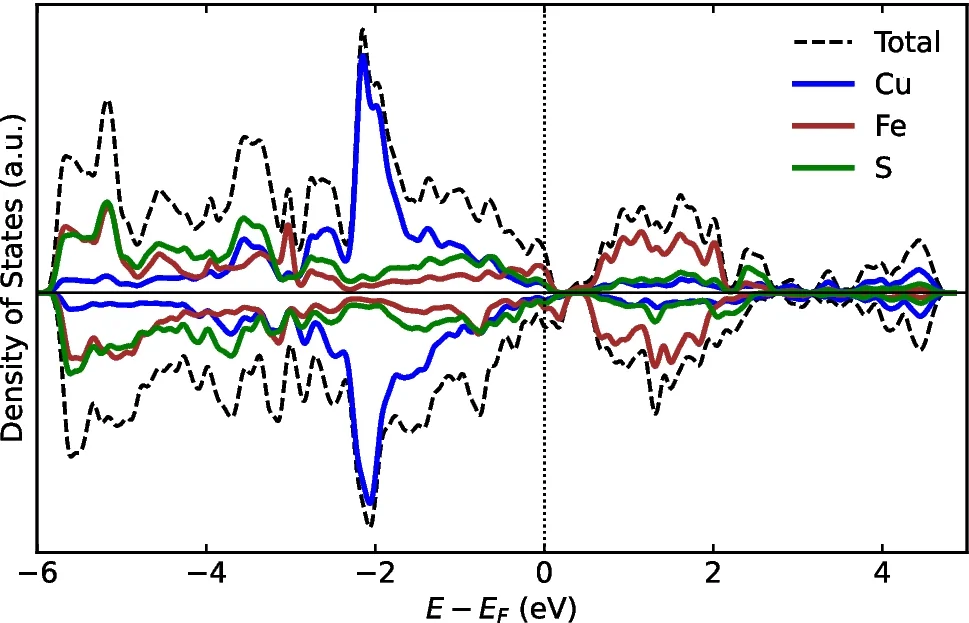
Total Density of States (DOS) of the (001)–S chalcopyrite surface. The vertical dashed line represents the Fermi level

Figure 2 illustrates the Density of States (DOS) for the (001)–S surface, presenting the total contribution of all electronic states. As shown in Fig. 2, iron-derived 3*d* states are predominantly located near the Fermi level, identifying Fe atoms as the primary active sites for chemical interaction with adsorbed species. This is consistent with reports indicating that both H$_{2}$O and O$_{2}$ preferentially adsorb onto iron sites [43, 55].

**Fig. 3:**
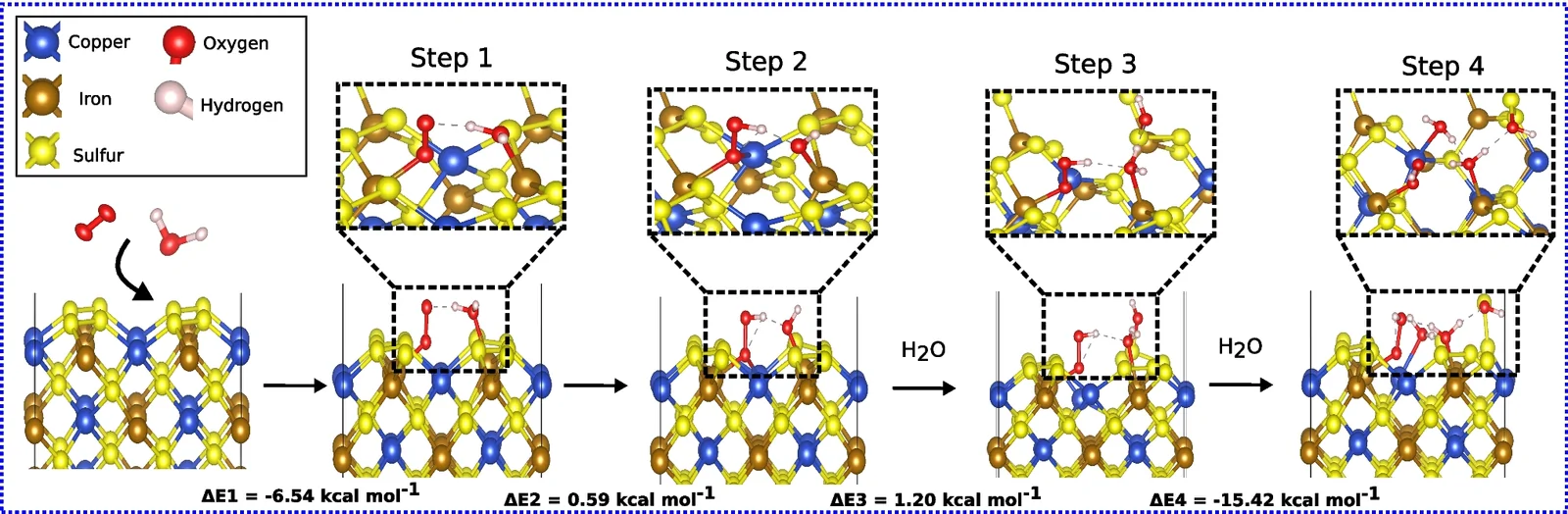
Proposed mechanism for the oxidation reactions on the (001)–S chalcopyrite surface

Several adsorption configurations were evaluated as starting points for the oxidation mechanism, including single-molecule adsorption of O$_{2}$ or H$_{2}$O and co-adsorption at different surface sites. The most thermodynamically stable configuration involves the simultaneous adsorption of O$_{2}$ and H$_{2}$O at neighboring iron sites, consistent with previous reports [43]. The overall mechanism comprises four sequential steps (Fig. 3): (i) co-adsorption of O$_{2}$ and H$_{2}$O on Fe sites, releasing −6.54 kcal mol$^{-1}$; (ii) proton transfer from H$_{2}$O to the O$_{2}$-derived intermediate, yielding Fe-OOH and Fe-OH species previously detected spectroscopically [50]; (iii) adsorption and dissociation of a second H$_{2}$O molecule, forming S$_{2}$-OH and regenerating an Fe-OH$_{2}$ environment; and (iv) H$_{2}$O adsorption on copper, which provides the largest stabilization of the series (−15.42 kcal mol$^{-1}$). The overall energy profile indicates a thermodynamically favorable process. Minor endothermic steps are observed along the pathway; however, since transition states were not explicitly computed in this work, their energetic barriers remain to be determined in future studies.

To provide a comprehensive understanding of each reaction step, electronic properties are analyzed through a combination of PDOS, Charge Density Difference maps, and Integrated Local Density of States (ILDOS). This approach allows us to correlate the energy-level shifts in the density of states with the spatial redistribution of electrons and the localization of specific electronic states along the oxidation pathway, allowing site-specific charge transfer events to be connected to the overall surface reactivity.

Under simulated leaching conditions, chalcopyrite exhibits a cooperative reaction mechanism in which metallic centers and adsorbates interact in a coupled manner rather than independent fashion. The initial stages are dominated by Fe-centered activation of O$_{2}$, whereby electron donation from Fe into antibonding π* orbitals of O$_{2}$ promotes its activation. In contrast, H$_{2}$O adsorption induces a weaker direct electronic perturbation but plays a decisive role in enabling proton transfer and structural rearrangement. The overall surface reactivity therefore emerges from the interplay between site-specific charge transfer at Fe, subsequent electron redistribution across the adsorbate–surface interface, and progressive involvement of Cu and S sites in later stages of the mechanism.

As a reference, Table 1 compiles the Bader charges and PDOS integrals for the relevant species in the gas phase (O$_{2}$, H$_{2}$O, and OH) and on the clean chalcopyrite surface sites (Fe$_{1}$, Fe$_{2}$, Cu, and S). The evolution of these quantities along the four-step reaction pathway is summarized in Table 2, providing a consistent electronic description of how charge transfer governs surface reactivity at each stage, organized by specific active sites and their evolving chemical environments (e.g., Fe$_{1}$–O$_{2}$ ⟶ OOH).

**Table 1:** Bader charges (e) and PDOS integrals for surface atoms and gas-phase reference species

Site	Configuration	Bader (e)	PDOS
*Surface*			
Fe1	O$_{2}$ → OOH	1.01	6.55
Fe2	H$_{2}$O → OH → H$_{2}$O	1.01	6.55
Cu	H$_{2}$O adsorption	0.61	9.75
S	OH adsorption	−0.42	4.46
*Gas phase*			
O$_{2}$	isolated molecule	−0.01	3.84
H$_{2}$O	isolated molecule	−1.28	6.96
OH	isolated radical	−0.59	7.46

Only oxygen atoms involved in adsorbed species are considered in the Bader and PDOS analysis

**Table 2:** Bader charges (e) and PDOS integrals along the four-step reaction mechanism on the chalcopyrite surface

Site	Environment	Step 1		Step 2		Step 3		Step 4	
		Bader	PDOS	Bader	PDOS	Bader	PDOS	Bader	PDOS
Fe1	Fe1–O$_{2}$ → OOH	1.16	6.28	1.18	6.15	1.18	6.18	1.16	6.47
Fe2	Fe2–H$_{2}$O → OH → H$_{2}$O	1.14	6.47	1.20	6.45	1.10	6.45	1.11	6.47
Cu	Cu–H$_{2}$O	0.65	9.73	0.73	9.65	0.57	9.73	0.69	9.76
S	S–OH	−0.34	4.45	−0.29	4.37	0.72	3.80	0.87	3.79
*Adsorbate atoms*									
O–Fe1	O$_{2}$ → OOH	−0.40	4.35	−0.52	4.47	−0.52	4.49	−0.54	4.54
O–Fe2	proton transfer species	−1.26	5.01	−1.15	5.07	−1.66	4.99	−1.64	5.01
O–Cu	Cu–H$_{2}$O	−	−	−	−	−	−	−1.67	4.93
O–S	S–OH	−	−	−	−	−1.56	4.84	−1.52	4.81

The O$_{2}$ molecule adsorbed on Fe1 evolves into OOH from Step 2 onward. Water initially adsorbed on Fe2 follows a H$_{2}$O → OH → H$_{2}$O sequence via proton transfer to the O–O intermediate

**Fig. 4:**
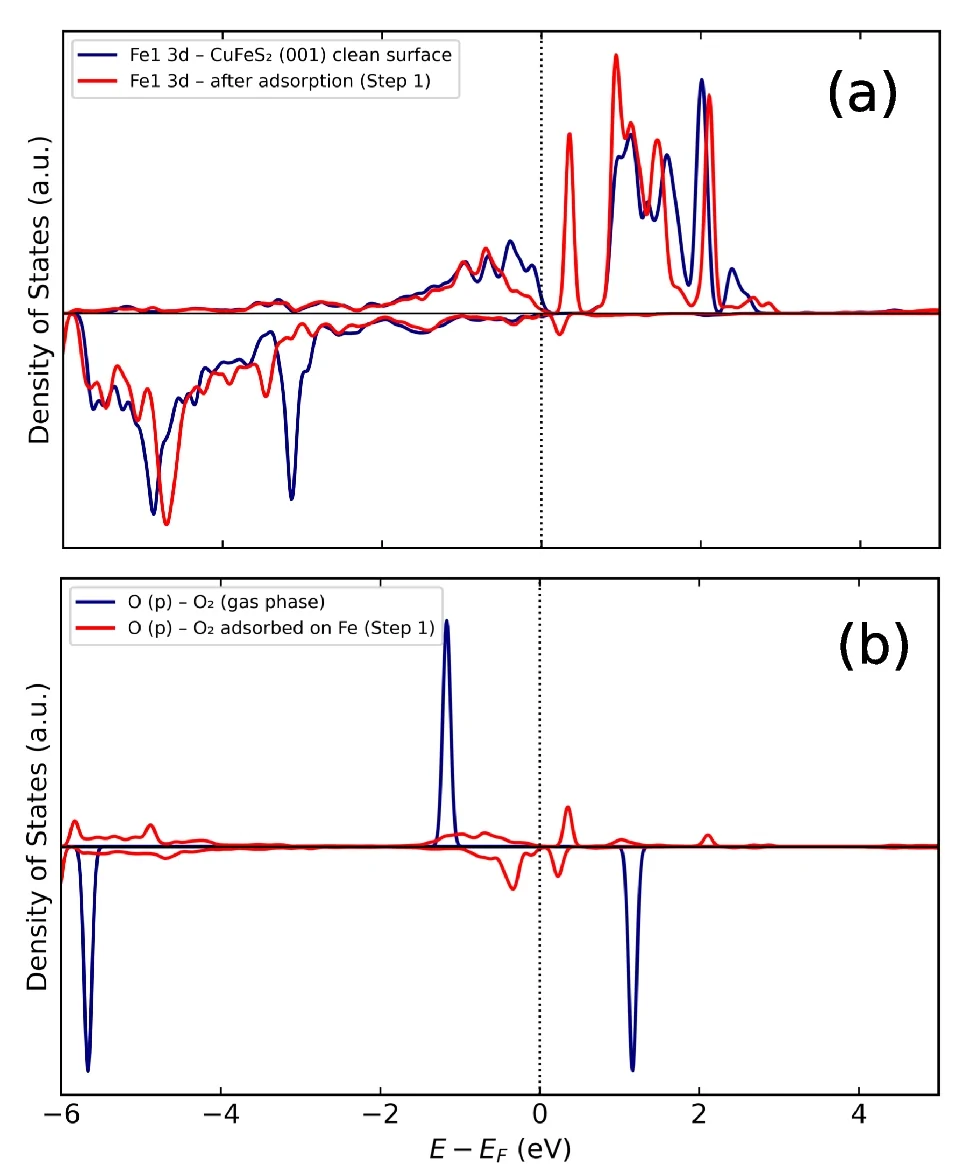
Projected density of states (PDOS) for atoms involved in the first step of the mechanism: **a** Fe site where O$_{2}$ is adsorbed and **b** O atom of the adsorbed O$_{2}$

The first step involves the co-adsorption of O$_{2}$ and H$_{2}$O at distinct Fe sites. O$_{2}$ adsorption proceeds via charge transfer from the Fe site to the molecule. The Fe Bader charge increases from 1.01 *e* to 1.16 *e*, while the Fe 3*d* PDOS occupation decreases from 6.55 to 6.28, consistent with electron donation into the antibonding $\uppi ^*$ orbitals of O$_{2}$. This charge transfer activates the molecule, elongating the O-O bond from 1.23 (gas phase) to 1.34 Å, a value characteristic of superoxide (O$_{2}{}^{-}$) species on transition metal surfaces [67], and confirming partial reduction of the adsorbed molecule. This mechanism is visually confirmed by charge density difference maps (Fig. 6a), Fe site exhibits a clear vertical polarization, with charge depletion (blue) at its base and accumulation (yellow) at the top facing the adsorbate. A matching pattern of depletion (blue) at the lower binding interface and accumulation (yellow) at the upper exposed region is observed for both oxygen atoms along the O$_{2}$ molecule. This directional charge distribution tracks the electron flow from the surface into the molecule, confirming its chemical activation. PDOS analysis (Fig. 4) shows extensive overlap between the Fe 3*d* and O 2*p* states, indicating orbital hybridization and the disappearance of gas-phase-like features as the Fe−O−O complex is formed.

**Fig. 5:**
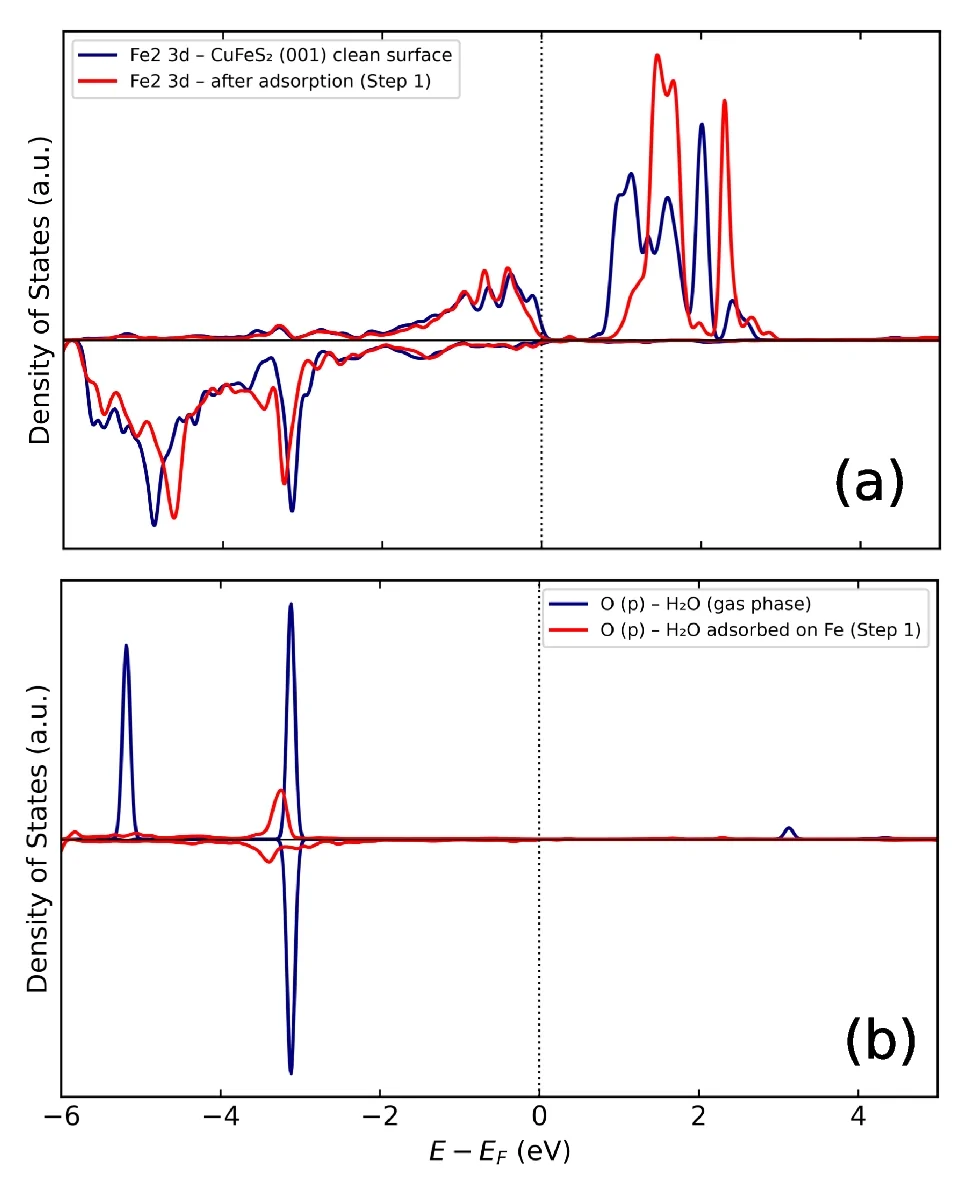
Projected density of states (PDOS) for atoms involved in the first step of the mechanism: **a** Fe site where H$_{2}$O is adsorbed and **b** O atom of the adsorbed H$_{2}$O

**Fig. 6:**
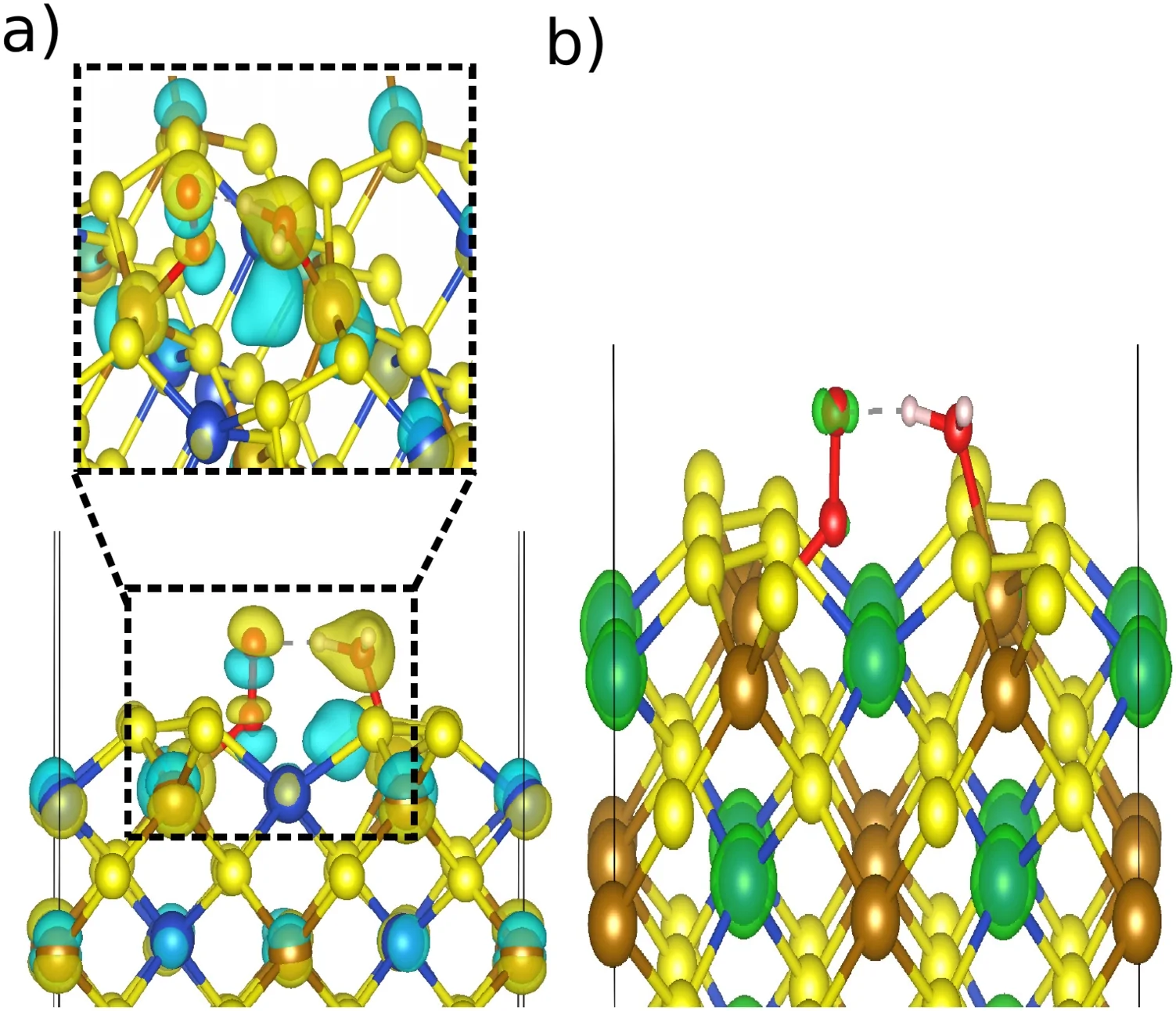
**a** Charge density difference map for the first step of the reaction mechanism, highlighting electronic redistribution upon adsorption. Yellow and blue isosurfaces indicate charge accumulation and depletion, respectively. **b** ILDOS (Region 2) for the First Stage. Isosurfaces represent the density of states near the Fermi level ($E_f$). Green circles highlight surface atoms where non-local electronic response and charge redistribution induced by the adsorbates occur

H$_{2}$O adsorption results in a weaker perturbation. The Fe Bader charge increases to 1.14 *e*, with a minor decrease in Fe 3*d* occupation (6.55 to 6.47). The O atom in H$_{2}$O shows negligible charge variation (−1.28 *e* to −1.26 *e*), although its PDOS integral decreases substantially (6.96 to 5.01). These results indicate that adsorption is dominated by electronic redistribution rather than net charge transfer. As shown in Fig. 5, the PDOS of both iron and water oxygen exhibit only minor shifts upon adsorption. However, the maintenance of the characteristic gas-phase-like peaks for H$_{2}$O alongside a clear energy overlap with the Fe 3*d* states confirms orbital hybridization through coordination. This is consistent with the role of water as a polarizable species that interacts without molecular activation. Charge density difference maps (Fig. 6a) visually confirm these distinct binding features. The H$_{2}$O-bound Fe site behaves similarly to the O$_{2}$ adsorption, showing a clear split with depletion (blue) at the base and accumulation (yellow) at the top. For the adsorbate, however, the H$_{2}$O molecule is characterized by a continuous electronic accumulation (yellow lobe) that forms a cohesive cloud encompassing the H-O-H framework. This uniform accumulation highlights that H$_{2}$O acts as a polarizable ligand without undergoing the localized electronic disruption observed for O$_{2}$.

ILDOS analysis (Fig. 6b) reveals that electronic states near the Fermi level are localized predominantly on the terminal O atom of the Fe-O-O group, indicating this site as the most reactive region of the adsorbate intermediate. No significant ILDOS contribution is observed for the H$_{2}$O molecule, consistent with its electronic stability at this stage. Although Fe acts as the primary electron donor, its contribution near the Fermi level is negligible after binding, as the Fe valence electrons are redistributed into the Fe-O bond and the O$_{2}$ $\uppi ^*$ states. The resulting electronic localization at the terminal oxygen creates favorable conditions for proton transfer from the neighboring H$_{2}$O, initiating surface hydroxylation and setting the stage for Fe–OOH and Fe–OH formation in Step 2.

The initial stage of the process involves the first oxidation step, leading to partial oxidation at the iron sites. Subsequently, the transfer of H to the FeOO species yields FeOOH. The Fe sites responsible for O$_{2}$ activation continue to donate electron density in this step, as evidenced by the decrease in 3*d* PDOS occupation (from 6.28 to 6.15) and a subtle rise in Bader charges (1.16 *e* to 1.18 *e*). The second Fe site, associated with H$_{2}$O dissociation, displays a distinct electronic response: its Bader charge increases (1.14 e to 1.20 e), while the PDOS occupation remains nearly constant but undergoes an upward shift toward the Fermi level, reflecting a redistribution of 3*d* states rather than net electron loss.

**Fig. 7:**
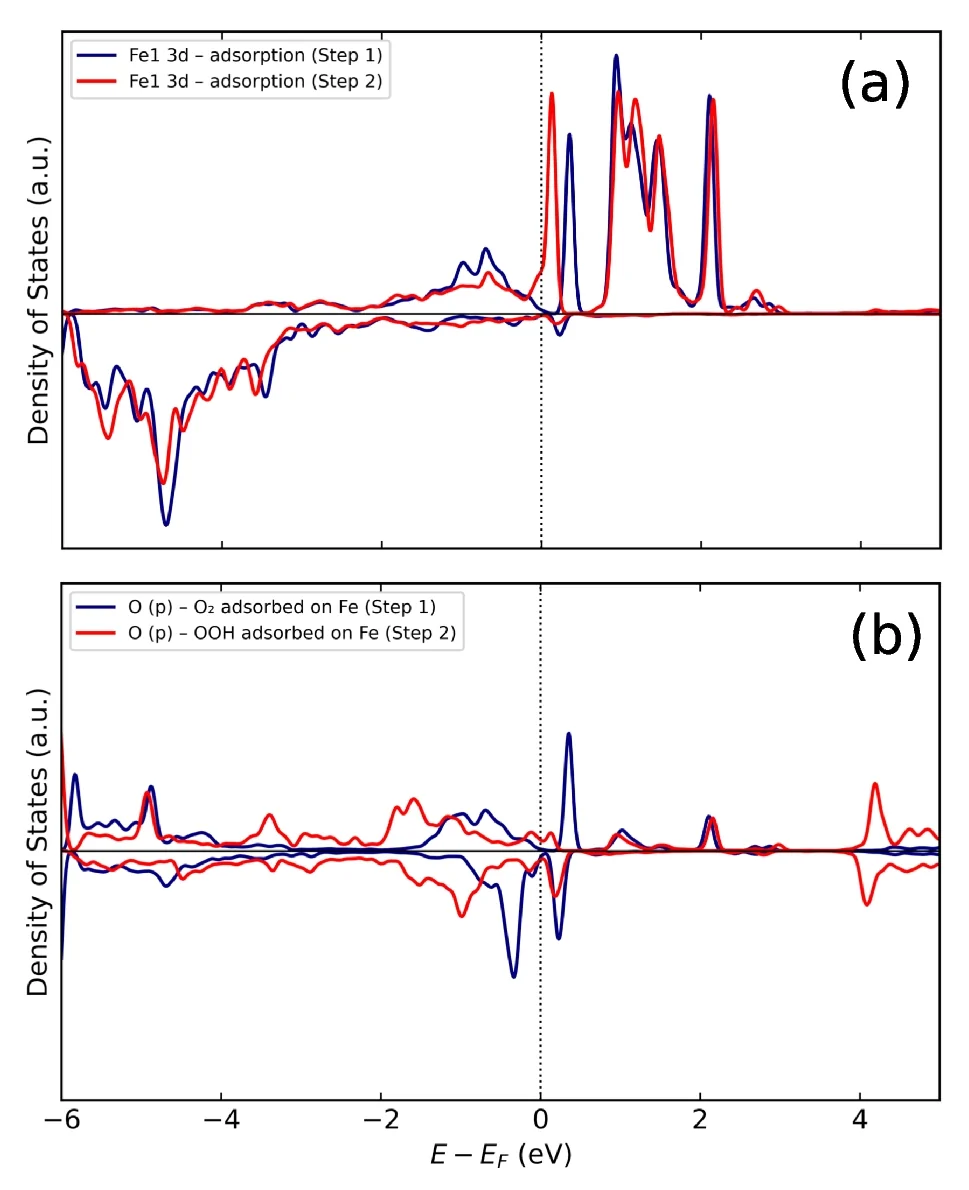
Projected density of states (PDOS) for atoms involved in the second step of the mechanism: **a**) Fe site where OOH is adsorbed and **b**) O atom of the adsorbed OOH

**Fig. 8:**
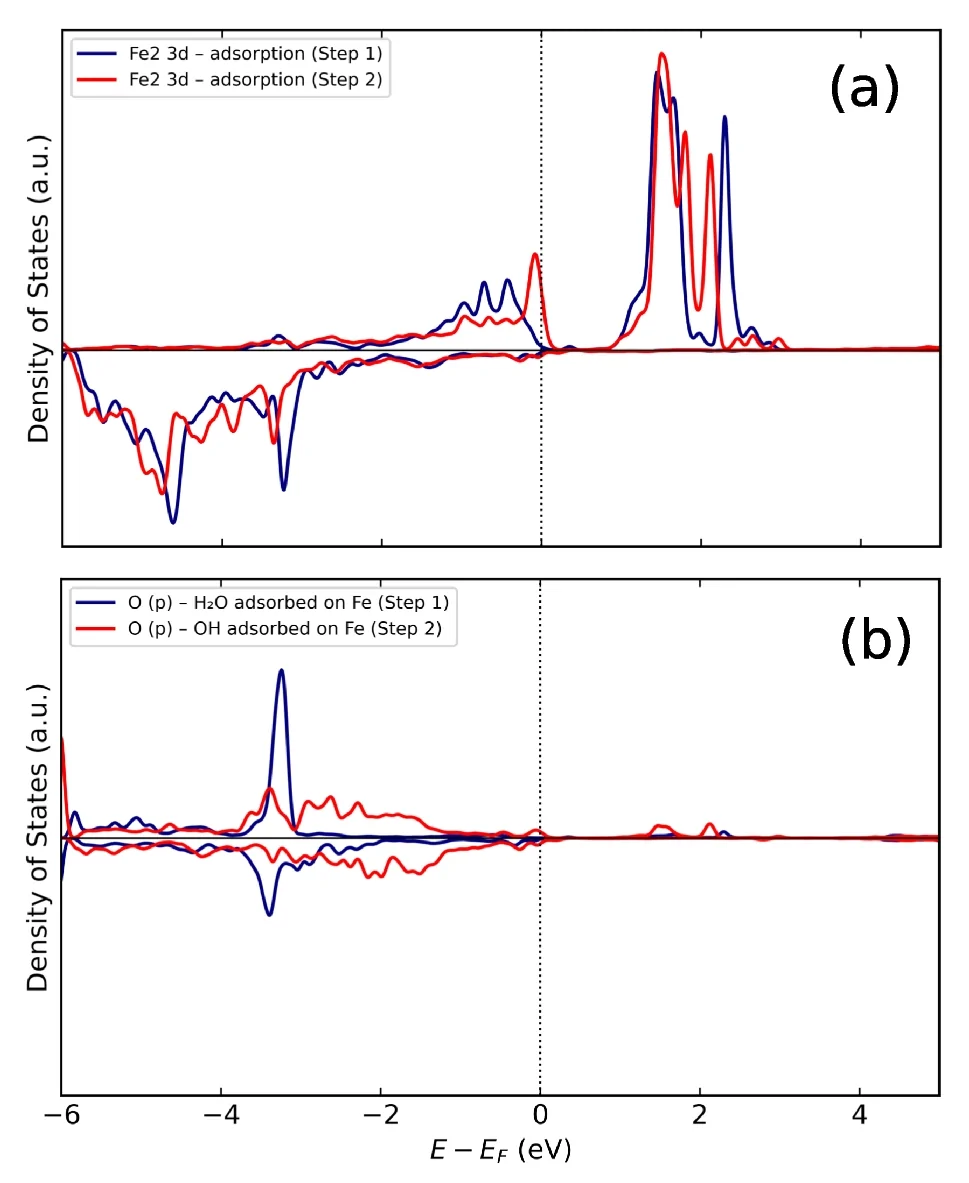
Projected density of states (PDOS) for atoms involved in the second step of the mechanism: **a**) Fe site where OH is adsorbed and **b**) O atom of the adsorbed OH

The evolution of individual oxygen atoms provides insight into this mechanism. In the Fe-OOH complex, the oxygen bonded to the iron center gains electron density (−0.40 *e* to −0.52 *e*) with minimal changes in PDOS, signaling the progressive activation of the O-O bond. In contrast, the oxygen in the Fe-OH species exhibits local electronic redistribution, with its Bader charge shifting from (−1.26 *e* to −1.15 *e*) and increased occupation of the PDOS, which is consistent with the stabilization of the O-H bond. These results can be observed in Figs. 7 and 8, showing changes after the formation of oxygenated species.

**Fig. 9:**
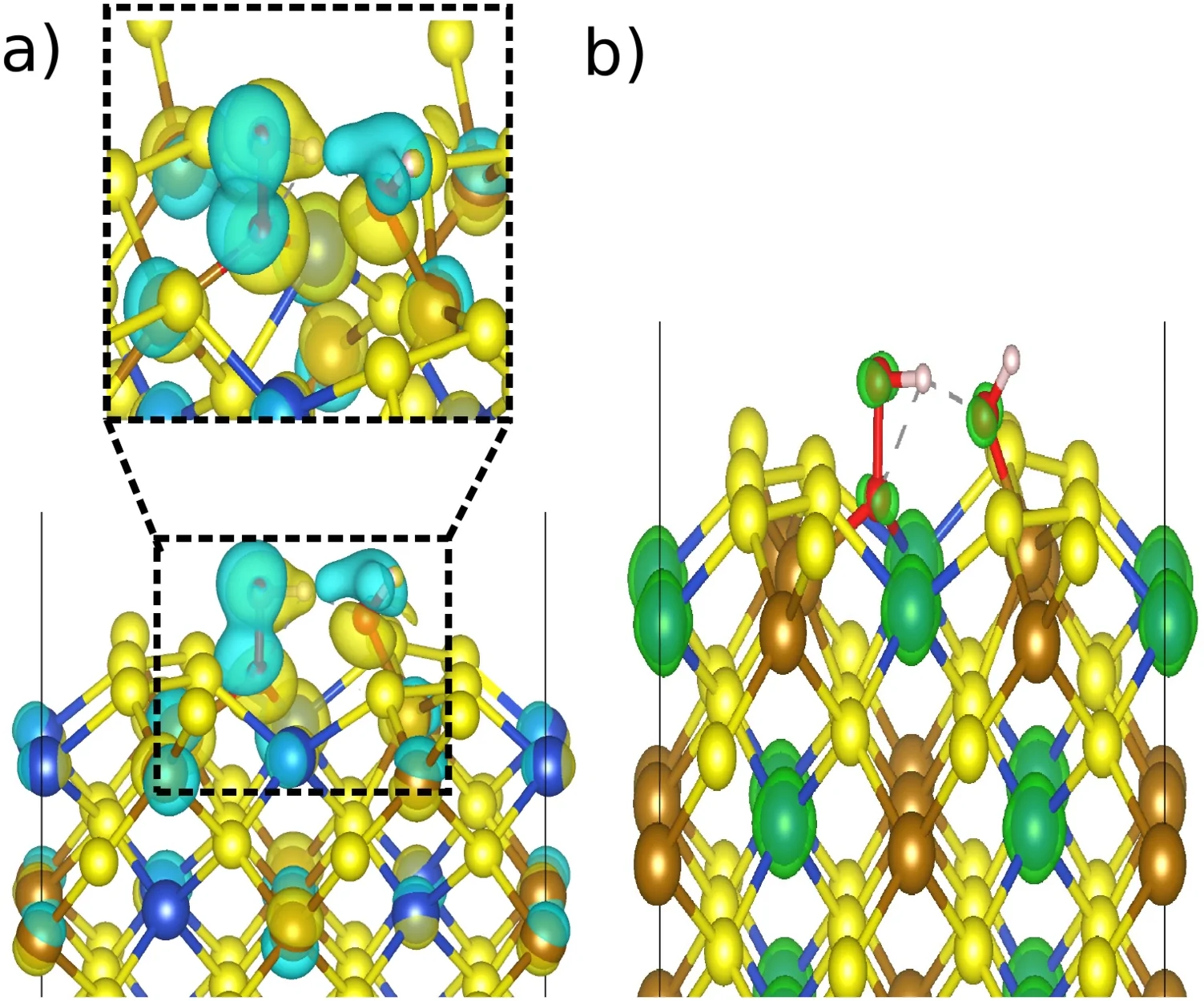
**a**) Charge density difference map for the second step of the reaction mechanism, highlighting electronic redistribution upon adsorption. Yellow and blue isosurfaces indicate charge accumulation and depletion, respectively. **b**) ILDOS (Region 2) for the Second Stage. Isosurfaces represent the density of states near the Fermi level ($E_f$). Green circles highlight surface atoms where non-local electronic response and charge redistribution induced by the adsorbates occur

As illustrated in Fig. 9a, charge density difference analysis provides a spatial visualization of the proton transfer mechanism. Within the newly formed OOH species, the oxygen atom that captures the proton exhibits a distinct region of charge depletion (blue lobe). Concurrently, a well-defined, vertically oriented electronic accumulation (yellow lobe) encompasses the migrated hydrogen atom, signaling the formation and stabilization of the new O-H bond. This localized redistribution is contrasted by a significant depletion zone (blue cloud) that remains over the residual OH fragment, visually confirming the proton migration path from the original water molecule. Furthermore, the ILDOS analysis (Fig. 9b) suggests that states near the Fermi level are localized on these intermediates, marking their transition into electronically active species.

**Fig. 10:**
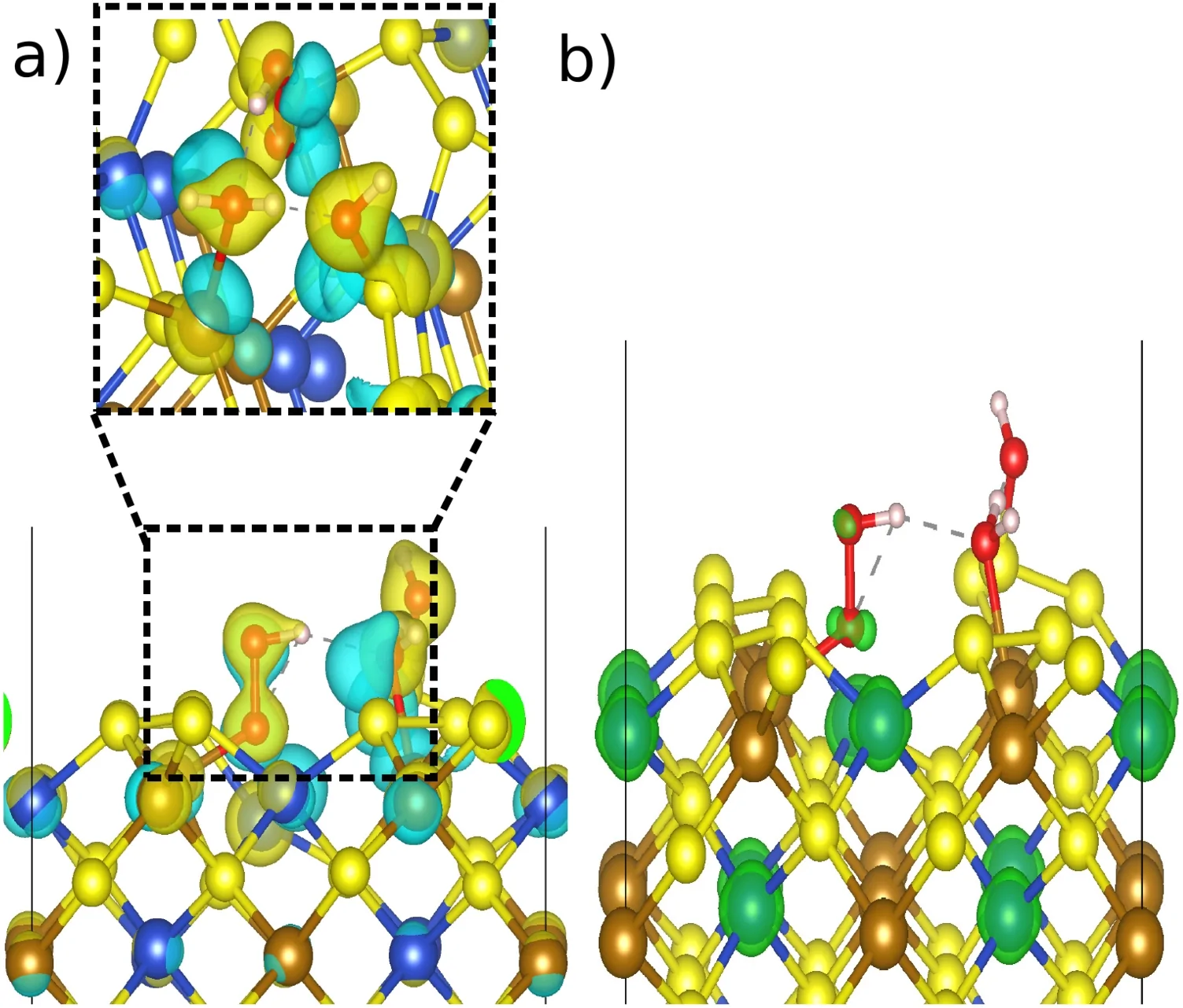
**a**) Charge density difference map for the third step of the reaction mechanism, highlighting electronic redistribution upon adsorption. Yellow and blue isosurfaces indicate charge accumulation and depletion, respectively. **b**) ILDOS (Region 2) for the Third Stage. Isosurfaces represent the density of states near the Fermi level ($E_f$). Green circles highlight surface atoms where non-local electronic response and charge redistribution induced by the adsorbates occur

In the subsequent stage, a second H$_{2}$O molecule adsorbs and dissociates into OH and H species. The OH group binds to a sulfur atom within the S−S dimer, while the H atom interacts with an Fe−OH site, resulting in an Fe−OH$_{2}$-like configuration. Charge density analysis (Fig. 10a) reveals electron accumulation in the S−OH region and depletion in the surrounding sulfur environment; this highlights the activation of sulfur sites and a clear break in local electronic symmetry. Furthermore, ILDOS analysis (Fig. 10b) shows that states near the Fermi level are primarily localized on the copper site and the OO moiety of the FeOOH species, with little contribution from the sulfur framework and the remaining surface sites. This result indicates that copper and the oxygenated intermediate play a major role in the electronic activity of the interface at this stage.

These electronic changes are accompanied by pronounced structural rearrangements: The S−Cu bond elongates from 2.35 to 3.62 Å, indicating effective bond rupture and displacement of the sulfur atom toward a more exposed surface configuration. The formation of S$_{2}$OH species represents the onset of sulfur oxidation and constitutes a plausible precursor for the subsequent generation of sulfate and other oxidized sulfur intermediates.

**Fig. 11:**
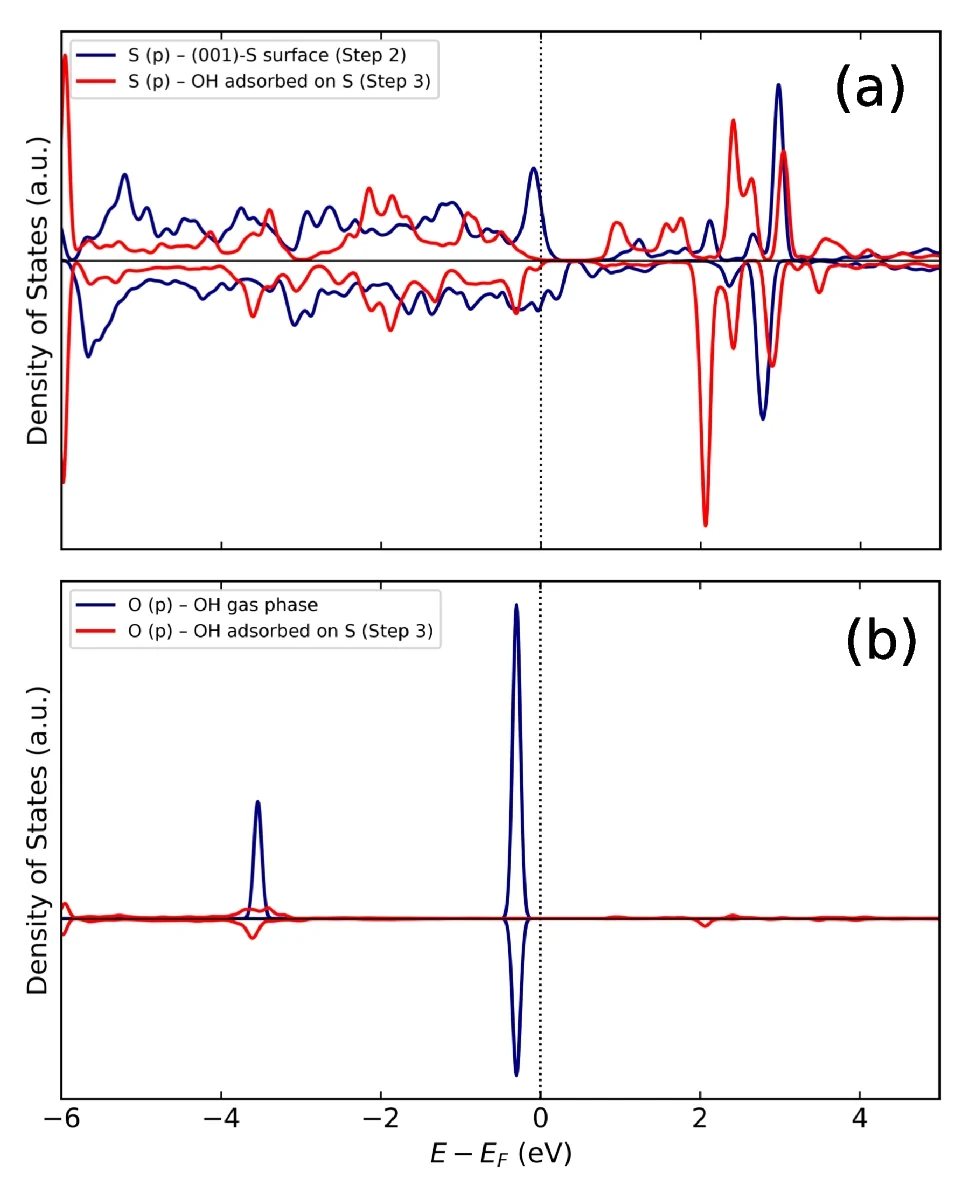
Projected density of states (PDOS) for atoms involved in the third step of the mechanism: **a**) S site where OH is adsorbed and **b**) O atom of the adsorbed OH

The sulfur atoms also exhibit notable electronic changes, with the Bader charge increasing from −0.29 *e* to 0.72 *e*, indicating loss of electron density. This behavior is consistent with the reduction in PDOS occupation from 4.37 to 3.80, reflecting depletion of sulfur electronic states. As shown in Fig. 11, the energy shifts observed after OH adsorption support the partial oxidation of sulfur during this mechanism. The disappearance of the oxygen-related peak after adsorption is also consistent with the formation of S$_{2}$OH species.

**Fig. 12:**
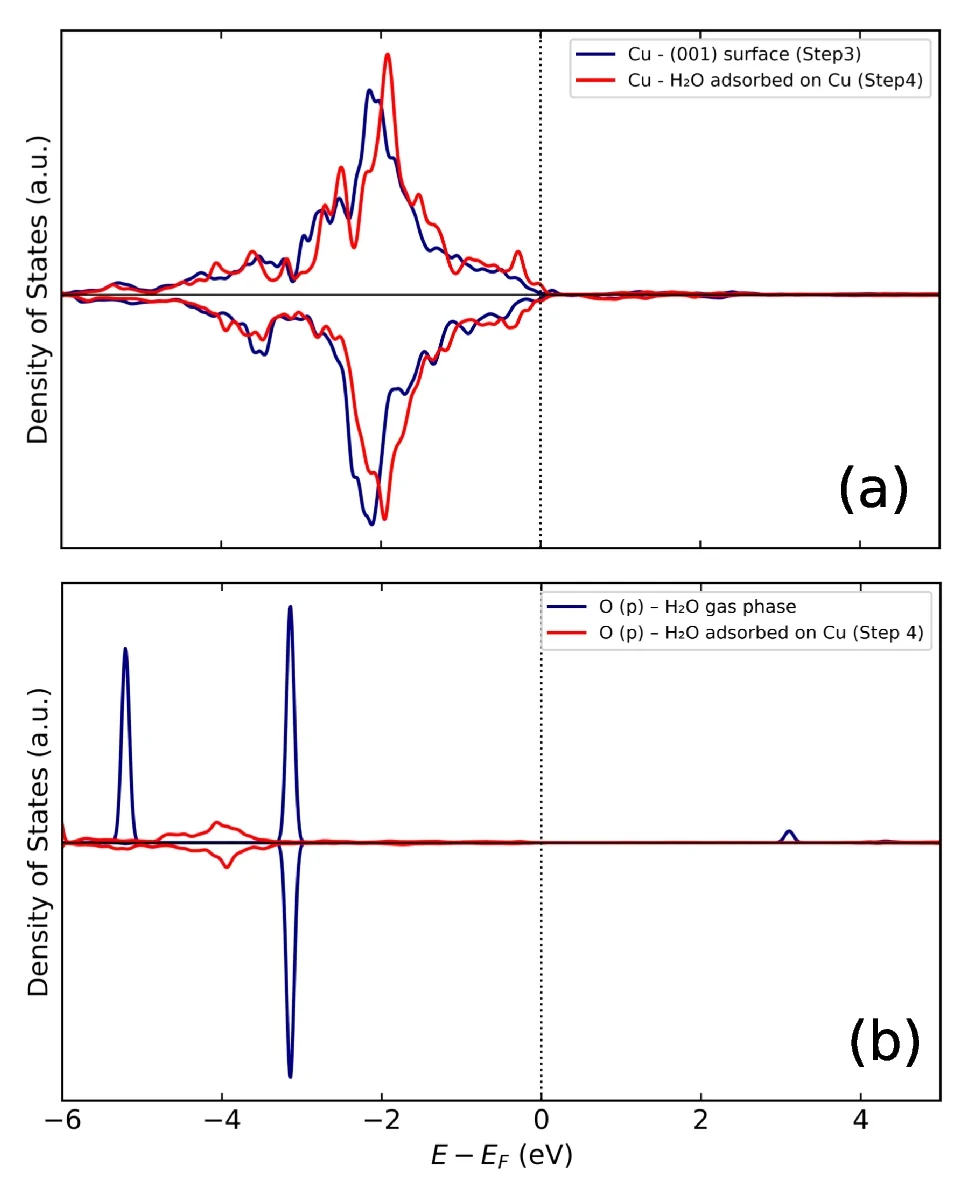
Projected density of states (PDOS) for atoms involved in the fourth step of the mechanism: **a**) Cu site where H$_{2}$O is adsorbed and **b**) O atom of the adsorbed H$_{2}$O

In the final stage, copper displays an electronic response distinct from those of Fe and S. Its Bader charge increases from 0.57 *e* to 0.69 *e* upon H$_{2}$O adsorption, while the *d*-band PDOS increases (9.73 to 9.76). More significantly, the PDOS (Fig. 12) shows an upward shift of the Cu *d*-states toward the Fermi level after adsorption, with the appearance of occupied states in that energy region, which underscores the active role of copper in the electronic redistribution that follows S−S bond rupture.

**Fig. 13:**
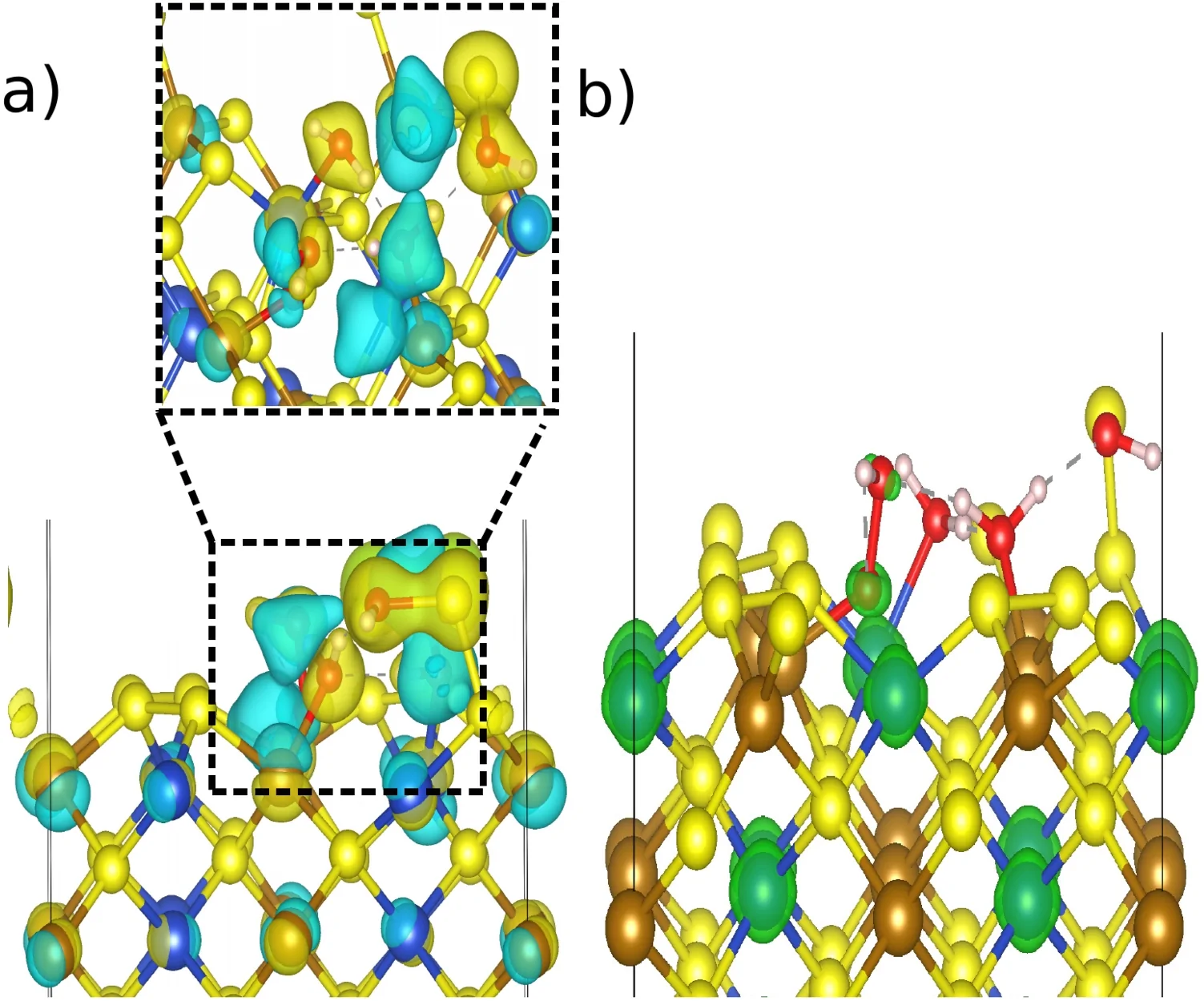
**a**) Charge density difference map for the fourth step of the reaction mechanism, highlighting electronic redistribution upon adsorption. Yellow and blue isosurfaces indicate charge accumulation and depletion, respectively. **b**) ILDOS (Region 2) for the Fourth Stage. Isosurfaces represent the density of states near the Fermi level ($E_f$). Green circles highlight surface atoms where non-local electronic response and charge redistribution induced by the adsorbates occur

Charge density difference and ILDOS analyses (Fig. 13a and b, respectively) further support this interpretation. The charge density difference maps display prominent regions of charge accumulation (yellow lobes) localized on the adsorbates, contrasted by a distinct charge depletion (blue lobes) concentrated at the underlying interfacial regions. This accumulation/depletion pattern reveals a clear charge redistribution from the surface metallic centers toward the chemical species at the interface, consistent with the formation of stable interfacial bonding states. Near the Fermi level, the ILDOS highlights localized states mainly on the O−O moiety of the Fe−OOH species and on the copper atom. In deeper energy regions, charge accumulation becomes more broadly distributed over both the adsorbates and the metallic centers.

The electronic response of the adsorbing water molecule further corroborates this interfacial behavior. Upon adsorption, the oxygen atom of the H$_{2}$O molecule undergoes a substantial increase in its Bader charge (from −1.28 *e* to −1.67 *e*), accompanied by a pronounced decrease in its PDOS occupation (from 6.96 to 4.93). This apparent contrast quantifies a severe local polarization, where the net electron gain is driven by a profound orbital reconfiguration to establish the interfacial bond. Ultimately, these quantitative shifts align with the charge density difference and ILDOS maps, confirming that copper actively mediates the electronic redistribution across the interface at this final stage of the mechanism.

It is important to emphasize that the model adopted in this work was designed to describe the interatomic interactions among the species involved in the oxidation process of chalcopyrite. Although the presence of multiple water and oxygen molecules may quantitatively affect the adsorption energies, the qualitative trends observed in this study are expected to be preserved.

## Conclusion

The four-step pathway traced here shows that oxidation of the chalcopyrite (001)-S surface is not a single event centered on one metal, but a sequence that moves from Fe to S to Cu, leaving a distinct electronic signature at each stage. O$_{2}$ adsorption on Fe opens the sequence: the O-O bond stretches from 1.23 to 1.34 Å and the Fe Bader charge rises from 1.01 *e* to 1.16 *e*, consistent with electron donation into the antibonding $\uppi ^*$ orbitals of O$_{2}$. Proton transfer from a neighboring H$_{2}$O molecule then produces Fe−OOH and Fe−OH, species that match those detected spectroscopically on leached chalcopyrite surfaces [50]. The third step moves the reaction away from iron: a second H$_{2}$O molecule dissociates on the S−S dimer, the S−Cu distance stretches from 2.35 to 3.62 Å, and the sulfur Bader charge shifts from −0.29 *e* to 0.72 *e*. This is where the sulfur framework begins to break apart, pointing to a plausible route toward the sulfate and polysulfide species reported in chalcopyrite passivation layers [23, 35]. Copper, largely inactive up to this point, only becomes electronically engaged in the fourth step, when H$_{2}$O adsorption pulls its *d*-states toward the Fermi level and raises its Bader charge to 0.69 *e*.

Iron being oxidized and stabilized first agrees with the well established experimental observation that Fe leaches out of chalcopyrite ahead of Cu [23]. The involvement of Cu and S in the later steps suggests that the passivating layer is not a single uniform phase, but a mix of electronically coupled environments, consistent with the long-standing difficulty in assigning it one clear composition [25].

The present model has two limitations worth stating plainly. Kinetic barriers between the four steps were not calculated, so the energetics reported here are thermodynamic only, and transition-state calculations would be needed to assess how accessible each step actually is. The surface was also treated with a small number of explicit water molecules, one or two per step, rather than a full aqueous environment. This is a common simplification in DFT studies of mineral-water interfaces, but it means the adsorption energies should be read as a first approximation: additional water molecules, hydrogen-bonding networks, and solvent screening could shift the energetics, even though the qualitative sequence of Fe, S, and Cu activation is expected to hold. Extending this pathway with more explicit solvent, or with implicit solvation models, is a natural next step toward a more quantitative picture of chalcopyrite passivation under real leaching conditions.

## Data Availability

No datasets were generated or analysed during the current study.
